# Serine Protease Inhibitors—New Molecules for Modification of Polymeric Biomaterials

**DOI:** 10.3390/biom10010082

**Published:** 2020-01-04

**Authors:** Katarzyna Szałapata, Monika Osińska-Jaroszuk, Justyna Kapral-Piotrowska, Bożena Pawlikowska-Pawlęga, Rafał Łopucki, Robert Mroczka, Anna Jarosz-Wilkołazka

**Affiliations:** 1 Department of Biochemistry and Biotechnology, Maria Curie-Skłodowska University, Akademicka 19, 20-033 Lublin, Poland; monika.osinska-jaroszuk@poczta.umcs.lublin.pl (M.O.-J.); anna.wilkolazka@poczta.umcs.lublin.pl (A.J.-W.); 2 Department of Functional Anatomy and Cytobiology, Maria Curie-Skłodowska University, Akademicka 19, 20-033 Lublin, Poland; justyna.kapral-piotrowska@poczta.umcs.lublin.pl (J.K.-P.); bozena.pawlikowska-pawlega@poczta.umcs.lublin.pl (B.P.-P.); 3 Laboratory of X-Ray Optics, Centre of Interdisciplinary Research, The John Paul II Catholic University of Lublin, Konstantynów 1, 20-708 Lublin, Poland; lopucki@kul.pl (R.Ł.); rmroczka@kul.lublin.pl (R.M.)

**Keywords:** covalent immobilization, protease inhibitors, modification of biomaterials

## Abstract

Three serine protease inhibitors (AEBSF, soy inhibitor, α_1_-antitrypsin) were covalently immobilized on the surface of three polymer prostheses with the optimized method. The immobilization efficiency ranged from 11 to 51%, depending on the chosen inhibitor and biomaterial. The highest activity for all inhibitors was observed in the case of immobilization on the surface of the polyester Uni-Graft prosthesis, and the preparations obtained showed high stability in the environment with different pH and temperature values. Modification of the Uni-Graft prosthesis surface with the synthetic AEBSF inhibitor and human α_1_-antitrypsin inhibited the adhesion and multiplication of *Staphylococcus aureus* subs. *aureus ATCC^®^ 25923^TM^* and *Candida albicans* from the collection of the Department of Genetics and Microbiology, UMCS. Optical profilometry analysis indicated that, after the immobilization process on the surface of AEBSF-modified Uni-Graft prostheses, there were more structures with a high number of protrusions, while the introduction of modifications with a protein inhibitor led to the smoothing of their surface.

## 1. Introduction

Polymeric substances, which are used very often as biomaterials, are much more similar to human tissues than inorganic substances. They can be used in targeted therapies in which specific interactions between a biomaterial and patient’s cells are planned [1]. Synthetic polymers owe their popularity to such features as the ease of production, the ability to control the properties of the polymer, and the ability to manipulate the material easily [2]. Despite the development of more advanced biomaterials, prosthetic infections (in particular, vascular prostheses) are still a serious problem for implantology. The microorganisms that are most often responsible for causing prosthetic infections include among the others coagulase-negative staphylococci (30–43%), *Staphylococcus aureus* (12–23%), or Gram-negative rods (3–6%). Approximately 10–12% of infections are mixed infections and 10–11% are unknown [3,4,5]. Microorganisms living within the biofilm are characterized by a significant increase in invasiveness. It is estimated that as much as 65% of hospital infections are associated with biofilm formation [6]. Pathogenic microorganisms easily produce biofilms on surfaces such as catheters or surgical implants [7]. Unfortunately, older people and patients diagnosed with rheumatoid arthritis, obesity, or diabetes are particularly vulnerable to the occurrence of post-operative infections after prosthesis implantation [8].

Among the most popular methods of biomaterial modification to improve aseptic properties, the use of nanoparticles or silver ions, should certainly be mentioned [9,10,11]. Currently, vascular prostheses modified by antibiotics are also used very often. Primarily, gentamicin, amikacin, or rifampicin are used in these modifications, and the antimicrobial effect of such prostheses is much more efficient than in the case of silver ions and nanoparticles [12,13,14,15,16,17,18]. Unfortunately, the use of metal ions and various types of antibiotics is one of the most problematic issues in implantology because it can lead to the development of drug resistance mechanisms. Therefore, it seems reasonable to conduct research on the use of new antimicrobial substances, including proteolytic enzyme inhibitors that will be significantly more effective in the fight against infections within implanted prostheses [19,20,21].

In the above-mentioned organisms, we can distinguish a number of proteolytic enzymes, which are classified as virulence factors. For example, SPATEs family in *Escherichia coli* [22]; SspA, SplA-F, SspB, ScpA, and Aur in *Staphylococcus aureus* [23]; SAP (secreted aspartyl proteases) in *Candida* sp. [24]; or LasA and LasB elastases in *Pseudomonas aeruginosa* [25]. Therefore, the inhibition of proteases action through the use of proteolytic enzyme inhibitors (in particular those with a broad spectrum of activity) can definitely contribute to decreased viability of dangerous microorganisms and can help fight back prostheses infections in their early stages. Especially, when protease inhibitors rapidly bind to the active site of microbial proteases, can block them and instantly form an irreversible or partially-reversible compound, which halts the digestion of human tissues [26,27,28].

To address this situation, protease inhibitors have been proposed as new substances with antimicrobial potential. Inhibitors of proteolytic enzymes have natural biological activities, e.g., antiviral, antibacterial, antiparasitic, or antitumor effects [29]. Thanks to many years of research, protease inhibitors have gained the status of registered drugs and have been successfully used in the treatment of diseases caused by HIV, influenza, or HCV virus [30,31,32]. Currently, many biologically active substances covalently immobilized on the surface of biomaterials are known [9,12,13,14,15,16,17,33,34,35]. Therefore, we decided to perform covalent immobilization of three serine protease inhibitors, one synthetic (AEBSF) and two natural (soy inhibitor and α_1_-antitrypsin), on the surface of biomaterials that are commonly used in regenerative medicine. The justification for undertaking this new research topic is confirmed by reports on the use of protease inhibitors as substances that may affect the inhibition of inflammation within implanted catheters [36] or attenuates the prothrombotic properties due to the presence of a inhibitor layer covering the catheters [37].

The main goal of our work was to immobilize three serine protease inhibitors on the surface of various biomaterials commonly used in regenerative medicine. For this purpose, the process of immobilization optimization was carried out, taking into account the selection of an appropriate inhibitor concentration or the type and concentration of the cross-linking compound. The activity of immobilized inhibitors at different values of pH and temperature were studied. The influence of sterilization on the activity of the obtained preparations, the surface structure of modified prostheses, and their long-term stability were also tested. The aim of the final stage of the research was to examine the suitability of the modified biomaterials for inhibition of biofilm formation on their surface by the microbial reference strains (*E. coli ATCC^®^ 25922^TM^*, *P. aeruginosa ATCC^®^ 27853^TM^*, *S. aureus* subs. *aureus ATCC^®^ 25923^TM^*, and *C. albicans* from the collection of the Department of Genetics and Microbiology, UMCS).

## 2. Materials and Methods 

### 2.1. Inhibitors

Three serine protease inhibitors were used for covalent biomaterial modification: (a) 4-(2-aminoethyl)benzenesulfonyl fluoride hydrochloride (AEBSF)—a synthetic inhibitor; (b) a soy inhibitor isolated from *Glycine max* (SI)—a natural inhibitor of plant origin; and (c) α_1_-anitrypsin (α_1_-AT)—a natural human origin inhibitor. All inhibitors were supplied by Sigma Aldrich.

### 2.2. Carriers for Immobilization

Two groups of immobilization supports were used in the experiments (Table 1). The first part of the study was carried out on controlled pore glass (functionalized with 1% APTES solution in acetone) to select the optimal method of immobilization for each inhibitor and its storage conditions. The next stages of the experiments were carried out on biomaterials, which have different functional groups –OH, -COOH, or –NH_2_ thanks to the impregnation with gelatin or collagen (Table 1).

### 2.3. Covalent Immobilization of Protease Inhibitors

The controlled pore glass or biomaterial (approximately 1 g) was washed with distilled water and 0.1 M phosphate buffer pH 7.0. In the next step, the activation process was started, which proceeded as follows [38]:

(a) GLA—the carrier was suspended in a solution of GLA in 0.1 M phosphate buffer pH 7.0 and shaken for 1 h at room temperature. Then carrier was washed with 0.1 M phosphate buffer pH 7.0 until the GLA odor disappeared, reduced with solution of NaBH_4_, and again washed with cold 0.1 M phosphate buffer pH 7.0;
(b) CDI—carrier was washed with 0.1 M KH_2_PO_4_. Then carrier was suspended in CDI dissolved in 0.1 M KH_2_PO_4_ and shaken for 30 min at room temperature. After activation the carrier was washed with distilled water and cold 0.1 M KH_2_PO_4_;
(c) DVS—the carrier was washed with 1 M Na_2_CO_3_. Then carrier was suspended in DVS solution in 1 M Na_2_CO_3_ and shaken for 2 h at room temperature. After activation, the carrier was washed with small portions of distilled water to reach pH 8 and then cold 0.1 M Na_2_HPO_4_.

Then, the activated carrier was suspended in an inhibitor solution with an optimized concentration (5 mL of the inhibitor solution per 1 g of the carrier – AEBSF 0.6 mg/mL; SI 1.5 mg/mL; α_1_-AT 0.5 mg/mL) and shaken for 3 h in room temperature. Then, the preparation was stored at refrigerated temperature (+4 °C) for about 12 h.

After each immobilization process, the non-covalent bounded inhibitor molecules were removed by washing with the following solutions with different pH and ionic strength values: 0.1 M phosphate buffer pH 7.0, 0.5 M NaCl, 0.1 M phosphate-citrate buffer pH 5, and distilled water. The active groups remaining on the carriers were blocked by washing with 0.5 M Tris-HCl buffer (pH 7.5). The immobilized inhibitor preparations were intensively rinsed with 0.1 M phosphate buffer (pH 7.0).

During the optimization of the conditions of proteolytic enzyme inhibitor immobilization, an appropriate concentration of each inhibitor (AEBSF 0.6–2.4 mg/mL; SI 1–2 mg/mL and α_1_-AT 0.1–1 mg/mL), the type of the cross-linking compound (glutaraldehyde, GLA; divinylsulphone, DVS; and carbodiimide, CDI), and its concentration (from 2.5 to 10%) were selected.

### 2.4. Determination of Inhibition Activity

The activity of inhibitors was determined according to the Lee and Lin method [39] in glycine buffer (pH 9.5). Porcine pancreas trypsin (MP Biomedicals) was used as a model enzyme and a 1% hemoglobin solution (Sigma Aldrich) was used as a substrate. Measurement of inhibitory activity started with a 20-min pre-incubation of the inhibitor (in a native or immobilized form) with the enzyme at 37 °C. Subsequently, the substrate was added to the reaction mixture and, after 60 min of incubation at 37 °C, the reaction was stopped by addition of 5% trichloroacetic acid. The samples were centrifuged (10 min, 1200 RPM) and their absorbance was measured at 280 nm. For samples of the inhibitor immobilized on the surface of various biomaterials, a prosthetic fragment with a designated mass was added to the reaction mixture and then the inhibitor activity was calculated for 1 mg of the modified prosthesis.

The protein concentration was determined with the Lowry method [40], using bovine serum albumin as a standard. One trypsin unit (U) is equal to ΔA_280_ of 0.001 per minute with hemoglobin as a substrate at pH 9.5 and 37 °C. One inhibitor unit (IU) is the inhibitor activity that reduces the enzyme activity by 1 U.

### 2.5. Determination of the Immobilization Process Efficiency and the Specificity of Chemical Bonds Formed Between Inhibitors and Immobilization Carriers

After washing the carriers, each of the individual fractions was collected (after rinsing) and the concentration of the tested inhibitors was determined therein. The concentration of SI and α_1_-AT inhibitors was measured spectrophotometrically using an Eppendorf BioSpectrometer [37], and the concentration of the AEBSF inhibitor was measured with the capillary electrophoresis method using Agilent Technologies 7100 Capillary Electrophoresis. The separation was carried out in a silica capillary with a total length of 60 cm (50 cm to the detection window) and Ø 50 μm at 25 kV and a temperature of 20 °C. The samples were dosed hydrodynamically for 4 s and detection thereof was carried out at 230 nm. The separation was carried out in phosphate buffer (50 mM disodium hydrogen phosphate—Na_2_HPO_4_ × 12 H_2_O in MilliQ water pH 9.5) and the analysis was carried out for 4 min.

The immobilization efficiency (IE) for the tested inhibitors and carriers was calculated as the percentage of the total inhibitor amount bound onto the carrier. IE was calculated from the Formula (1).

(1) $$\mathrm{IE}=\frac{concentration\;of\;the\;inhibitor\;bounded\;to\;the\;carrier}{concentration\;of\;the\;inhibitor\;intended\;for\;immobilization\;x\;100\%}$$

Based on the measurement of the inhibitor concentration in the fractions after the washing process, the percentage of inhibitor molecules bounded covalently and non-covalently to the biomaterials surface was determined. All inhibitor molecules associated with the surface of the prosthesis after the immobilization process were considered as the 100% value.

### 2.6. Stability at Different Temperature and PH Values

The effect of incubation at different pH values on the native and immobilized inhibitor activities was studied using 0.1 M Theorell-Steinhagen buffer with a pH range from 2 to 12. Fragments of modified prostheses (dimensions 0.5 × 0.5 cm) were suspended in 1 mL of buffer with an appropriate pH value. The incubation lasted 24 h. After this time, the modified biomaterials were washed 5 times with 0.1 M phosphate buffer pH 7 and the inhibition activity of the modified biomaterials was measured. The effect of temperature was determined by 30-min incubation of samples (0.5 × 0.5 cm of prosthesis in 1 mL of 0.1 M phosphate buffer pH 7) in a temperature range of 20–50 °C. After the incubation, the samples were washed with the same buffer and their activity was measured according to the inhibition activity assay.

### 2.7. Stability during Long-Term Storage

The inhibition activity of the immobilized preparations was measured every 7 days. The preparations were suspended in 0.1 M phosphate buffer at pH 7 or in 0.1 M phosphate-citrate buffer at pH 5 and stored at refrigeration temperature (4 °C). Based on the results obtained, the optimal buffer for storage of the modified materials was selected.

### 2.8. Effect of Sterilization on Inhibition Activity of Modified Biomaterials

The effect of two different sterilization methods was analyzed. The first of the tested variants was thermal sterilization (121 °C, 15 min) and the second was sterilization with UV radiation (two cycles, each lasting 5 min). The 0.5 × 0.5 cm fragments of prostheses were sterilized and, after completion of sterilization, the inhibition activity of the modified biomaterials was measured and compared to the inhibition activity of non-sterilized biomaterials.

### 2.9. MIC and MBC Analysis

The analyses of the biological activity of the modified biomaterials were carried out on the following microorganisms: *Escherichia coli* (ATCC^®^ 25922^TM^), *Pseudomonas aeruginosa* (ATCC^®^ 27853^TM^), *Staphylococcus aureus* subsp. *aureus* (ATCC^®^ 25923^TM^), and *Candida albicans* (from the collection of the Department of Genetics and Microbiology, UMCS). A series of bacterial cultures (*E. coli*, *P. aeruginosa*, *S. aureus*) and a fungal culture (*C. albicans*) were prepared on Mueller–Hinton Bullion and Lactose Broth medium, respectively. The bacteria and yeasts grew with the addition of native protease inhibitors in the range of the following concentrations: 0.125–3 mg/mL for AEBSF and SI, and 0.06–1.5 mg/mL for α_1_-AT. All experiments were carried out according to the microdilution method developed by the Clinical and Laboratory Standards Institute [41,42].

### 2.10. Analysis of Biofilm Formation

Analysis of biofilm formation was carried out according to the modified method proposed by Brown et al. [43]. Samples of biomaterials (0.5 × 0.5 cm) were placed in sterile 6-well plates. In each well, there were 5 mL of Tryptic Soy Broth (TSB) with microbial cells (approximate density 10^5^ cfu/mL). The samples were incubated for 72 h at 37 °C and 130 rpm. Each day, the TSB medium was replaced with a fresh portion. At the end of the incubation, the prosthesis samples were washed with sterile PBS buffer, placed on sterile plates in 5 mL of fresh TSB medium, and 1% TTC solution was added. Then, the observation of the formation of red formazan was carried out. SEM preparations were first washed in 0.1 M PBS buffer pH 7.4, then fixed for 1 h in 4% GLA solution and again washed with 0.1 M PBS buffer pH 7.4. Then the prostheses samples were dehydrated in ethanol solutions (25, 50, 70 and 100% EtOH) and placed in a desiccator for 24 h before applying a gold layer. SEM micrographs were taken (TESCAN VEGA 3 LMU) of variants that showed the greatest potential for inhibiting the growth of pathogenic microorganisms and biofilm production.

### 2.11. Analysis of Biomaterial Surface Structure Using Optical Profilometry

The surface structure of materials that had the highest antimicrobial potential in the earlier stages of the study was analyzed. The experiment was carried out using a non-contact optical profilometry technique (Veeco WYKO NT 9800 profilometer). The objective lens magnification was set to 20×, which corresponds to a 0.48 µm sampling (pixel) size. The processing of the images (*n* = 20) was carried out in the SPIP 5.1.3 program, where 8 parameters of the morphological structure of biomaterials (Sds, Sq, Ssk, Sku, Sdq, Sz, Ssc, and Sdr) were analyzed. The data were statistically analyzed using the Kruskal–Wallis nonparametric test in the XLStat program.

### 2.12. Statistical Analysis

All the results are expressed as mean ± SD from three experiments (*n* = 3). The data were analyzed using one-way ANOVA followed by a post hoc Tukey test. Values of *p* ≤ 0.05 only were reported as statistically significant.

## 3. Results

The first stage of the research was based on the development of optimal immobilization conditions for three serine protease inhibitors. At each stage of optimization process, we examined the efficiency of immobilization and inhibition activity after the immobilization process, which was crucial for choosing optimal parameters. In the initial optimization studies, glass with controlled porosity was used as a model carrier for immobilization. In the case of the synthetic inhibitor AEBSF, the selection of the optimal cross-linker compound was omitted since the only functional group that could participate in the formation of the covalent bond with the carrier is the -NH_2_ group. Table 2 summarizes all the optimal immobilization parameters that were analyzed during the entire optimization process. For all inhibitors, GLA was selected as the optimal cross-linking compound. However, its concentration used in the biomaterial activation process was differentiated depending on the serine protease inhibitor tested and ranged from 2.5 to 5%. Preparations stored in the optimal buffer solutions and at refrigeration temperatures retained on average 50% of their initial activity. However, the most stable formulation was the immobilized synthetic inhibitor AEBSF, which retained 75.6% of the initial activity after one month of storage.

After the development of the optimal immobilization conditions for each of the tested inhibitors, a number of immobilization processes were carried out to determine the basic parameters of the newly obtained modified medical materials (Table 2). In the case of the synthetic AEBSF inhibitor, one of the highest immobilization efficiencies was obtained for the Uni-Graft prosthesis (55.5%). It was also characterized by the highest activity of the immobilized AEBSF inhibitor (31.8 IU/mg of carrier) and the very high proportion of covalent bonds (93.7%) in inhibitor binding to the prosthesis surface. The lowest immobilization yield was obtained for the Codubix prosthesis (41.7%), which also showed the lowest activity of the bounded AEBSF inhibitor (2.1 IU/mg of carrier). In the case of SI, the highest immobilization efficiency was recorded for the Hemagard prosthesis (51.3%). The highest activity was noticed for the modified Uni-Graft prosthesis (52.2 IU/mg of carrier), for which the highest percentage of covalent bonds was found. The lowest activity and immobilization efficiency was again observed in the case of the Codubix prosthesis. For human α_1_-AT, the lowest immobilization efficiency was achieved on the surface of the Uni-Graft prosthesis (9.1%); nevertheless, it was characterized by the highest activity of the bound inhibitor (19 IU/mg of carrier). The highest efficiency of α_1_-AT immobilization was noticed for the Hemagard prosthesis (25.9%). Additionally, it showed equally high inhibition activity (18.6 IU/mg of carrier) as the modified Uni-Graft prosthesis.

**Figure 1:**
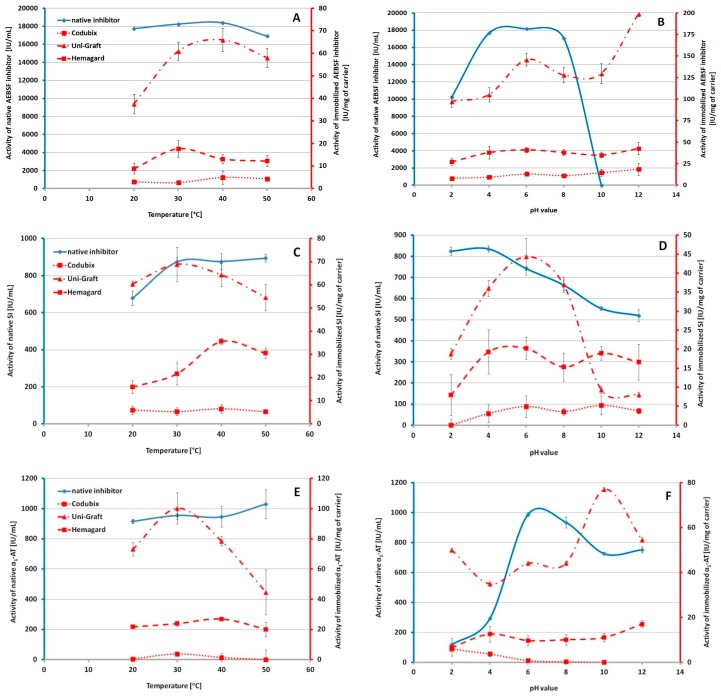
Inhibitor activity profiles after incubation in the environment with different values of temperature and pH—(**A**) and (**B**) AEBSF inhibitor; (**C**) and (**D**) SI; (**E**) and (**F**) α_1_-AT.

A very important parameter of biologically active biomaterials is their stability in different temperature and pH conditions. In addition, it is also important to maintain high activity of biologically active materials in the human body. A slight decrease in the activity at temperatures above 40 °C was observed for the native AEBSF inhibitor (Figure 1A). In the case of the AEBSF inhibitor immobilized on the surface of biomaterials, we observed an increase in the activity in the range of 30–40 °C. The largest decline in the activity was noted for the Uni-Graft prosthesis at 20 and 50 °C. Figure 1B shows the stability of the native and immobilized inhibitor AEBSF in various pH values. It was observed that the native AEBSF inhibitor rapidly lost its activity in solutions with pH values over 8. A high decrease in its activity was also observed at acidic pH values. In the case of the AEBSF inhibitor molecules immobilized on the surface of the tested prostheses, high inhibitor activity was observed in a wide spectrum of pH values. For the AEBSF inhibitor immobilized on the surface of the Uni-Graft prosthesis, two points of an activity increase were observed—at pH 6 and pH 12 (Figure 1B).

The native SI showed an increase in the activity at high temperature (Figure 1C). The highest activity was found at 30 °C for the Uni-Graft prostheses and at 40 °C for the Hemagard prosthesis. In addition, it was observed that the native SI was more active at acidic pH than at a pH value around 7 (Figure 1D). The SI inhibitor immobilization on the surface of the Hemagard prosthesis increased the inhibitor activity in two points—in the pH values 4–6 and 10. Soy inhibitor immobilized on the Codubix prosthesis increased the inhibitory activity in the pH values from 6 to 10, while the SI immobilized on the Uni-Graft prosthesis had the highest activity at pH 6, which is similar to other results.

The analysis of the different stability profiles of native and immobilized α_1_-AT showed that the temperatures influenced significantly the activity of the native inhibitor (Figure 1E). Native α_1_-AT also had higher activity in the acid medium than in the alkaline one, while the optimal pH values for this inhibitor are values close to pH 6–7 (Figure 1F). For immobilized α_1_-AT, we observed the greatest sensitivity to temperature changes (especially for the inhibitor immobilized onto the Uni-Graft prosthesis). The maximal activity of α_1_-AT immobilized on the surface of this biomaterial was observed for preparations incubated at 30 °C. Immobilization of α_1_-AT on the surface of the Hemagard prosthesis yielded a more stable preparation in a wide spectrum of pH values. In contrast, the inhibitor immobilized on the surface of the Codubix prosthesis completely lost its activity at a pH value higher than 6. We also observed a significant increase in the activity of α_1_-AT immobilized onto the Uni-Graft prosthesis after incubation at pH 10.

**Figure 2:**
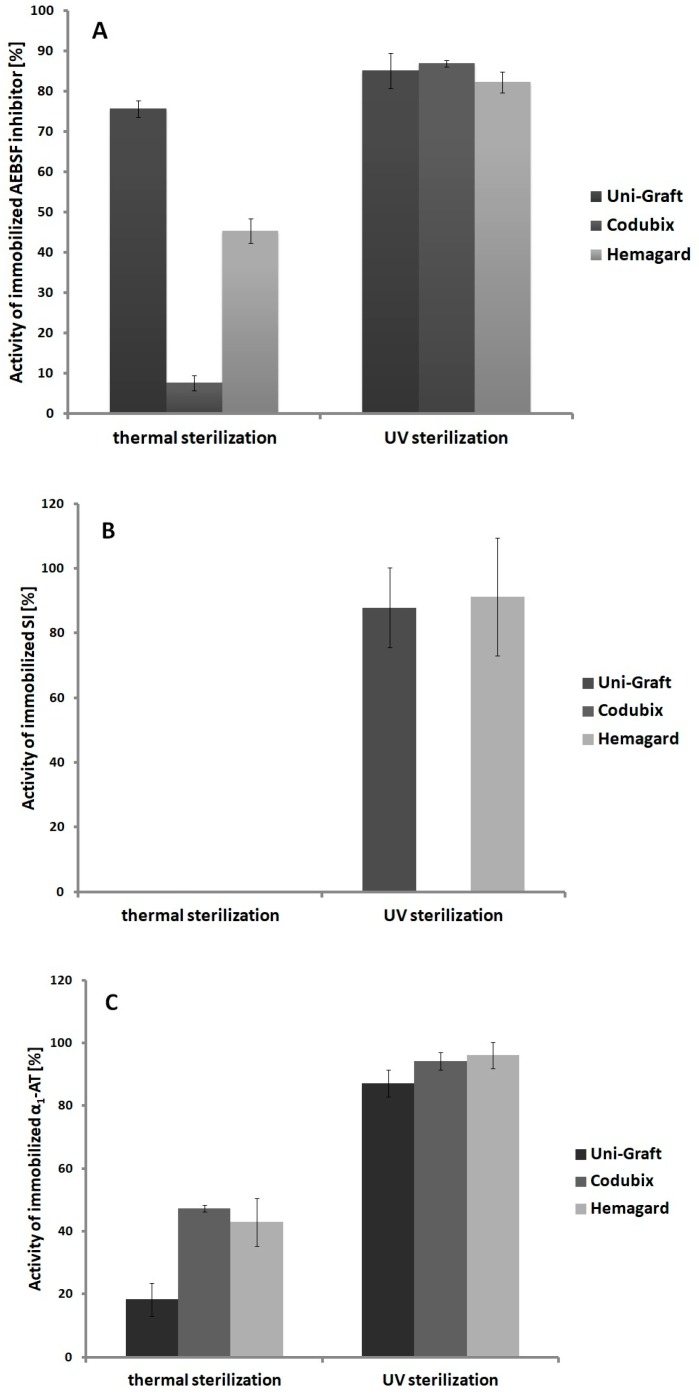
Influence of sterilization method on the activity of (**A**) AEBSF, (**B**) SI, and (**C**) α_1_-AT immobilized on various polymeric biomaterials.

The UV-radiation sterilization caused a slight decrease in the inhibition activity of the AEBSF-modified biomaterials (Figure 2A). All of the tested variants retained over 80% of their activity. However, after thermal sterilization, there was a significant decrease in the activity of the AEBSF inhibitor immobilized on the surface of all biomaterials. The AEBSF-modified Codubix prosthesis was the most sensitive to thermal sterilization (7.6% of the initial activity), while AEBSF-modified Uni-Graft prosthesis was the most resistant (75.6% of the initial activity). Thermal sterilization of the SI-modified prostheses caused complete inactivation of their inhibitory properties (Figure 2B). A similar dependency was observed in the case of the SI-modified Codubix prosthesis treated with the UV sterilization. The UV sterilization of the α_1_-AT-modified prostheses did not cause a high decrease in their inhibitory activity (Figure 2C). All the preparations remained active in the range of 87.2–96.1% of the initial activity. However, a large decrease in the inhibition activity was noticed in the case of the thermally sterilized α_1_-AT-modified prosthesis samples. The modified Uni-Graft prosthesis was the most sensitive to this type of sterilization (21.1% of initial activity), whereas the modified Codubix prosthesis exhibited the lowest sensitivity (47.3%).

The highest inhibition potential was observed for the AEBSF inhibitor against the *Pseudomonas aeruginosa* strain, where an inhibitor concentration above 0.5 mg/mL inhibited the growth of this microorganism (Table 3). In the case of the *Escherichia coli* and *Staphylococcus aureus* strains, a concentration of 2 mg/mL of the AEBSF inhibitor is required to inhibit their growth. The MBC analysis showed that the concentration of AEBSF above 3 mg/mL was bactericidal against the *Staphylococcus aureus* and *Pseudomonas aeruginosa* strains, and the concentration above 4 mg/mL was effective against the *Escherichia coli*. In the case of the two other inhibitors tested (SI and α_1_-AT), no bactericidal or fungicidal properties were found (Table 4). Due to the similar MIC and MBC values for the AEBSF inhibitor, a bactericidal effect is more likely expected on the tested microorganisms.

**Figure 3:**
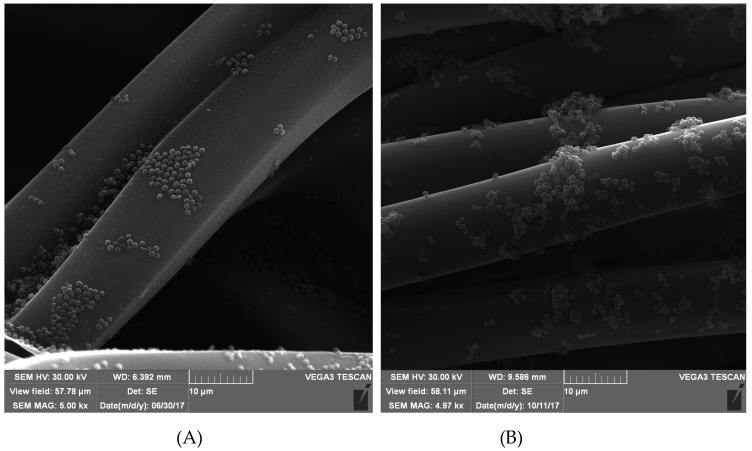
SEM photomicrographs of the Uni-Graft prosthesis surface (**A**) modified with AEBSF inhibitor and (**B**) unmodified, after incubation with *Staphylococcus aureus* cells

**Figure 4:**
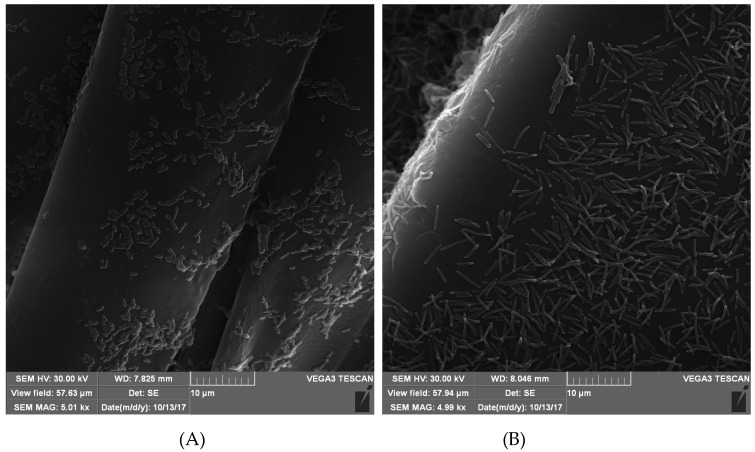
SEM photomicrographs of the Codubix prosthesis surface (**A**) modified with α_1_-AT and (**B**) unmodified, after incubation with *Candida albicans* cells.

**Figure A1:**
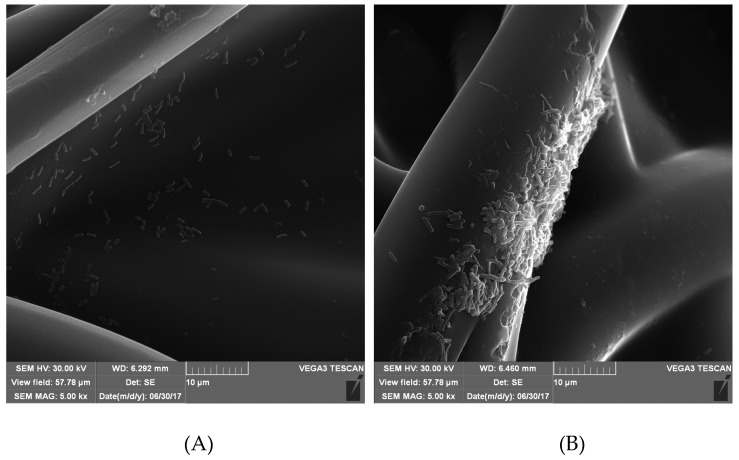
SEM photomicrographs of the Uni-Graft prosthesis surface (**A**) modified with α_1_-AT and (**B**) unmodified, after incubation with *Candida albicans* cells.

**Figure A2:**
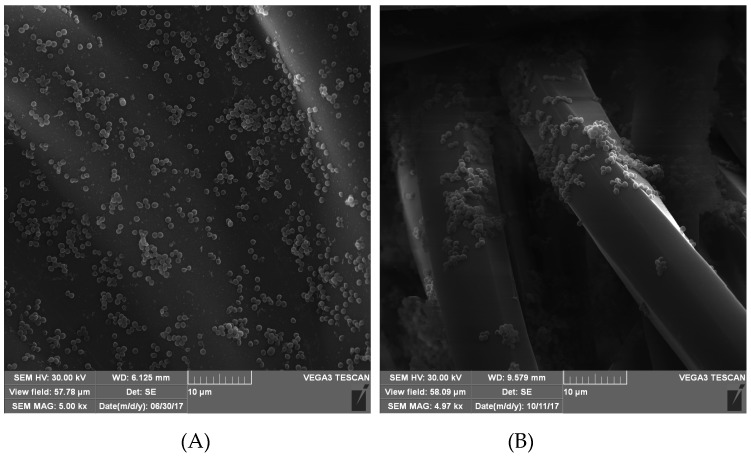
SEM photomicrographs of the Uni-Graft prosthesis surface (**A**) modified with α_1_-AT and (**B**) unmodified, after incubation with *Staphylococcus aureus* cells.

In the case of the Uni-Graft prosthesis modified with the AEBSF (Figure 3), SI, and α_1_-AT (Figure A1 and Figure A2), it was noticed that covalent modifications may favor the dispersion of bacterial cells on the surface of the prosthesis, while the occurrence of numerous dense microcolonies was observed on fragments of unmodified prostheses. Furthermore, the antimicrobial activity of the AEBSF inhibitor may have an additional bactericidal effect on *S. aureus* cells. The microscopic observation of the α_1_-AT-modified Codubix (Figure 4) and Hemagard prostheses showed that the presence of this compound inhibited the transformation of *Candida albicans* cells into a more invasive filamentous form. The covalent modifications of the prosthesis surface by the tested proteolytic enzyme inhibitors reduce adhesion of pathogenic microorganisms and thanks to it slowing down biofilm formation.

**Figure 5:**
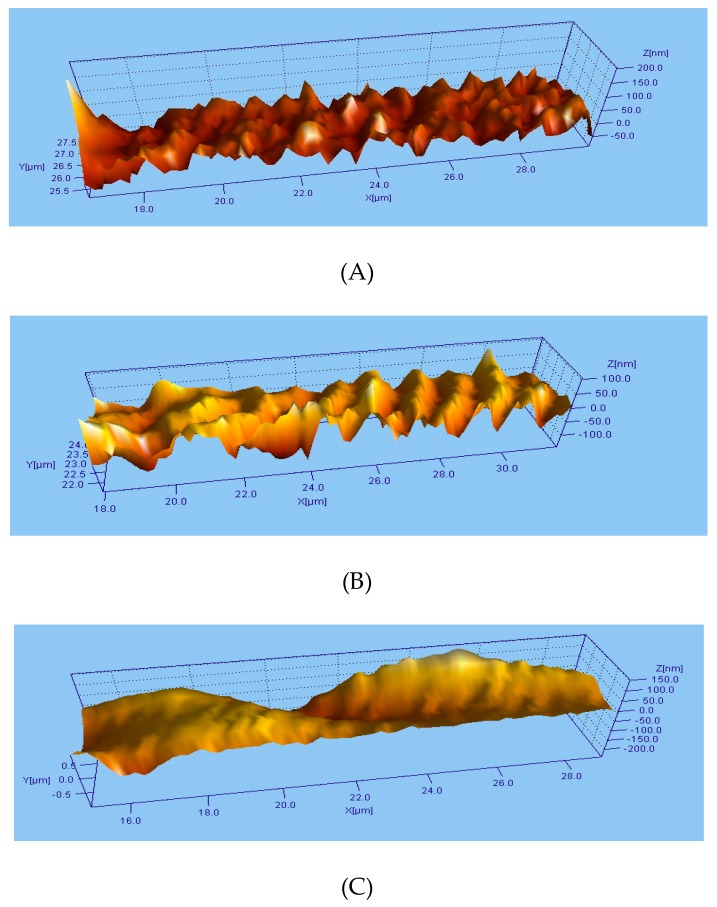
3D surface structure of the (**A**) unmodified, (**B**) AEBSF-modified, and (**C**) α_1_-AT-modified Uni-Graft prosthesis.

Table 5 presents a summary of the average values of the studied parameters of the prosthesis structure (Table A1) with their standard deviations. Due to the high structural variability among the tested prostheses, we also performed a non-parametric Kruskal–Wallis test, which demonstrated that the three examined prostheses differ from each other in the Sds parameter (describing the number of elevations on the prosthesis surface in mm^2^) in a statistically significant manner (*p* <0.0001). Moreover, the analysis of the Ssc parameter did not confirm that the introduction of the covalent changes in the surface structure of the examined prostheses had a negative impact on their sensitivity to plastic deformation. The analysis of the AEBSF-modified Uni-Graft prosthesis images (Figure 5) indicates the presence of more structures with high protrusions number (Figure 5B). This type of modification resulted in an increase of the peaks height, which were presented on the biomaterial surface. An increase in amplitude between the highest and the lowest points on the surface of the prosthesis was also observed (data not showed). On the other hand, the covalent modifications of the structure of polymeric prostheses with a protein inhibitor lead to the smoothing of their surface (Figure 5C). The presence of immobilized α_1_-AT on the surface of the Uni-Graft prosthesis caused the disappearance of small peaks and increased homogeneity of the biomaterial surface.

## 4. Discussion

Vascular prosthesis infections affect approximately 2–4% of the population of patients, but the frequency of their occurrence depends mainly on the area where the prostheses are located [44]. To reduce the risk of infection, various techniques of synthesis and modification of biomaterials are currently used. These include biomaterials made of substances with natural antimicrobial properties (like chitosan) or synthetic polymers (for example ePTFE), which reduce adhesion of pathogenic microorganisms to their surface, as well as coating the biomaterial surface with metal ions, antibiotics, or antimicrobial peptides [45,46,47,48,49]. Due to the fact that silver particles have a wide spectrum of activity, they negatively affect the development of both Gram-positive and Gram-negative bacteria. However, not only the high antimicrobial potential of silver ions encourages their frequent use in implantology. It has been proved that prostheses coated with silver acetate accelerates the vascularization process during the first 14 days after implantation, compared to unmodified prostheses [50]. Unfortunately, silver-coated polyester prostheses can also cause chronic inflammation, and the adverse side effect of this phenomenon is a decrease in their antimicrobial potential [51]. Currently, vascular prostheses modified with antibiotics (e.g., gentamicin, rifampicin, amikacin) are also very often used [15,16,17]. Unfortunately, the use of metal ions and various types of antibiotics is also one of the most problematic issues in implantology, because it can lead to the development of drug resistance mechanisms in pathogenic microorganisms. It is currently believed that bacteria are also capable of developing protective mechanisms acting against metal ion particles [52,53]. Therefore, it seems reasonable to conduct research on the use of new antimicrobial substances that are non-toxic and do not cause the development of resistance mechanisms.

As the first stage of the research, the optimization of immobilization of serine protease inhibitors on polymeric biomaterials was performed. For all inhibitor molecules, glutaraldehyde (GLA) was chosen as the optimal cross-linker. The use of this compound yielded the highest immobilization efficiency and activity of the immobilized inhibitors. Glutaraldehyde is one of the most commonly used cross-linking compounds in the industrial immobilization processes. The use of GLA molecules facilitates creation of a stable covalent bond between the binding biologically active substances and the immobilization matrix, whose surface is also enriched by the presence of amino groups (biomaterials were covered with gelatin or collagen coatings). In addition, in the case of protein inhibitors, a network of cross-links was created, which increased the structural stability of their molecules [54,55]. During the selection of the optimal concentration of the cross-linker, it was observed that, in the case of SI and α_1_-AT inhibitors, whose molecules have a large molecular mass compared to the AEBSF inhibitor, the use of a concentration of 2.5% GLA resulted in increased inhibition activity of the preparations. This phenomenon is probably related to the density of the inhibitor molecules on the surface of the carrier and the increased amount of created cross-links. By using lower glutaraldehyde concentrations, it is possible to provide free access of immobilized inhibitor molecules to target proteolytic enzymes. This also facilitates natural conformational changes within the inhibitor molecule that appear during its binding to the active site of the enzyme [56]. The highest activity of all three tested inhibitors was noticed on the Uni-Graft prosthesis. This is probably due to the gelatin coating present on the surface of the biomaterial, which has a naturally high waviness and allows the best possible exposure of inhibitory molecules in comparison to the other two prostheses (Codubix and Hemagard).

After completion of the first stage of the research, the physicochemical and biological properties of the optimized prosthesis preparations were studied. In the case of the inhibitor stability in the temperature range of 20–50 °C, small differences in the inhibitors activity were observed between the stability of the native molecules and the stability of the immobilized ones. Nevertheless, incubation of the immobilized inhibitor preparations in the studied temperature range did not affect their complete inactivation. On the other hand, during the incubation of the modified biomaterials at different pH values, the immobilization process significantly increased the stability of the native inhibitors, which are very sensitive to the action of the alkaline environment, causing their partial or complete inactivation. The most sensitive inhibitor was AEBSF, which was inactivated in the pH value above 8. Large differences in activity were observed between native inhibitors and those immobilized on the Uni-Graft prosthesis surface, what is probably caused by a change in the charge of inhibitor molecules in an alkaline environment and increase or decrease in their activity. The use of covalent immobilization for proteins or peptides and the formation of biologically active cross-links between their molecules during the immobilization process have a confirmed effect, enhancing conformational stability and thermostability [57]. In addition, our experiments have confirmed that the molecules of immobilized inhibitors retain high inhibitory activities in conditions similar to those that prevailing in the human body. These results suggest that the preparations of prosthesis modified with low molecular protease inhibitors (such as our model inhibitor, AEBSF) can be successfully used in regenerative medicine. Moreover, the maintenance of the high activity of preparations at 50 °C may indicate that they will be stable after sterilization with ethylene oxide, which takes place in this temperature range [58]. Despite the high thermal stability at 50 °C, some preparations lost their activity after sterilization. It was observed that all inhibitors were more or less sensitive to thermal sterilization and UV radiation sterilization. Thermal sterilization, unlike UV sterilization, causes irreversible thermal denaturation of protein inhibitors, while in the case of the synthetic AEBSF inhibitor it breaks down its molecule, what causes a complete loss of biological properties of protease inhibitors. However, for AEBSF inhibitor immobilized on the surface of the Codubix prosthesis, UV sterilization also had a critical effect on biological activity. This may be due to the lower humidity of the Codubix surface compared to the Uni-Graft and Hemagard prostheses, which are coated with gelatin and collagen, which can lead to faster breakdown of the AEBSF inhibitor molecule. Therefore, it seems justified to use sterilization with ethylene oxide or γ-radiation for the industrial sterilization of such modified biomaterials. These methods are currently widely used in the sterilization of various types of medical materials [58].

The most intensive antimicrobial activity of the modified biomaterials, which was manifested in anti-adhesive properties due to the presence of proteolytic enzyme inhibitors, was observed in the case of *S. aureus* and *C. albicans*. The mechanism of protease inhibitors action may be also related to blocking the activity of basic enzymes of metabolic pathways and proteins involved in essential physiological processes of microorganisms, which allow microbial cells to obtain nutrients and invade the host tissue [23,59,60]. Similar satisfactory effects inhibiting the adhesion and multiplication of bacterial cells were observed upon application of protein coatings with the Dhvar5 antimicrobial peptide and chitosan on the surface of titanium. The presence of a thin coating of antimicrobial substances resulted in reduction of *Staphylococcus aureus* adhesion to the surface of titanium up to 40% [61].

As part of the last task, the surface structures of the Uni-Graft prosthesis modified with the synthetic inhibitor AEBSF and α_1_-AT were compared. The most common methods for analysis of the surface structure and properties are based on the following techniques: SEM, AFM, XPS, SIMS, measurement of contact angle and ellipsometry, Raman spectroscopy, or profilometry [62]. Optical profilometry is a non-contact technique allowing analysis of the microgeometry of various materials (roughness and waviness) as well as macrogeometry, during which deviations are studied. In our research, depending on the inhibitor used in the covalent immobilization process, different micro- and macromorphological structure images of the modified prostheses were obtained. The results suggest that the use of biologically active substances of protein origin for biomaterial coating can reduce of surface roughness, which can be helpful in the design of materials with resistance to adhesion of pathogenic microorganisms. However, the use of small molecule inhibitors similar to AEBSF molecular weight can ensure greater freedom in the access of the active site of the inhibitor to the active site of the target enzyme. Preservation of the appropriate surface roughness and porosity of the biomaterial is, however, an important aspect during the colonization of implants by host cells (in example osteoblasts or fibroblasts) as well. Many studies prove that maintenance of the appropriate biomaterial roughness structure accelerates cell adhesion processes and increases biomechanical stability within the tissue covering the implant [63,64,65].

## 5. Conclusions

Conducting the several-stage process of covalent immobilization optimization helped to establish effective conditions for immobilization of three serine protease inhibitors on the surface of polymeric biomaterials. The immobilization process has a positive effect on the stability of the tested inhibitors. They show high activity in a wide range of pH and temperatures (only extreme pH values reduce their activity). The analysis of the biological activity against pathogenic strains has shown that biomaterials modified with AEBSF inhibitor may have anti-adhesive and bacteriostatic/batericidal properties, while biomaterials modified with protein inhibitors (SI and α_1_-AT) have rather anti-adhesive and dispersive properties. The highest activity was observed in the case of the Uni-Graft prosthesis modified with the synthetic inhibitor AEBSF and α_1_-AT against *S. aureus* and *C. albicans*. In addition, the covalent immobilization of proteolytic enzyme inhibitors on the surface of biomaterials introduces significant changes in their surface structure. Depending on the inhibitor used, the prosthesis surface may be smoother or rougher. Therefore, the use of proteolytic enzyme inhibitors seems to be a promising direction of research due to their natural biological activity and the ability to regulate the functioning of proteolytic enzymes of human and microbial origin.

## Figures and Tables

**Table 1:** Characterization of carriers modified by inhibitors of proteolytic enzymes.

Stage of Research	Carrier Type (Manufacturer)	Carrier Characteristics
**STAGE I** optimization process	Controlled pore glass (POCh)	pore diameter (D) – 25 nm pore volume (Vp) – 1.27 cm^3^/g specific surface of carrier (SHg) – 176.69 m^2^/g
**STAGE II** biomaterials functionalization	Uni-Graft^®^ K DV prosthesis (Braun Melsungen AG)	knitted gelatin-impregnated polyester prosthesis
	Skull bone prosthesis - Codubix (Tricomed)	polyester-polypropylene prosthesis with mechanical properties similar to natural bone
	Hemagard Intergard prosthesis (Maquet Getinge Group)	knitted polyester vascular graft impregnated with collagen

**Table 2:** Summary of optimal parameters of immobilization for three serine protease inhibitors and characterization of their activity, immobilization yield, and specificity of binding on the surface of studied polymer biomaterials.

Inhibitor	STAGE I—Optimization of Immobilization Process on CPG					STAGE II—Immobilization Process on Biomaterials Surface			
	Optimal Cross-Linking Compound	Optimal Concentration of Cross-Linking Compound	Optimal Concentration of Inhibitor	Optimal Storage Buffer	Stability during 1-Month Storage (%)	Biomaterial	Activity (IU/mg of Carrier)	Immobilization Yield (%)	Types of Bonds (%)
	GLA	5%	0.6 mg/mL	0.1 M phosphate buffer pH 7.0	75.6	Codubix	2.1 ± 0.5	41.7 ± 2.1	covalent – 87.6 non-covalent – 12.4
AEBSF						Uni-Graft	31.8 ± 1.5	55.5 ± 2.0	covalent – 93.7 non-covalent – 6.3
						Hemagard	5.58 ± 0.9	56.4 ± 1.7	covalent – 94.2 non-covalent – 5.8
SI	GLA	2.5%	1.5 mg/mL	0.1 M phosphate-citrate buffer pH 5.0	58.0	Codubix	12.6 ± 3.5	43.3 ± 1.6	covalent – 59.2 non-covalent – 40.8
						Uni-Graft	52.2 ± 6.8	43.9 ± 1.3	covalent – 89.0 non-covalent – 11.0
						Hemagard	26.3 ± 5.4	51.3 ± 2.1	covalent – 86.4 non-covalent – 13.6
	GLA	2.5%	0.5 mg/mL	0.1 M phosphate buffer pH 7.0	47.6	Codubix	5.0 ± 1.1	11.3 ± 0.9	covalent – 72.9 non-covalent – 27.1
α_1_-AT						Uni-Graft	19.0 ± 8.4	9.1 ± 0.7	covalent – 45.8 non-covalent – 54,2
						Hemagard	18.6 ± 2.2	25.9 ± 1.2	covalent – 65.3 non-covalent – 34.7

**Table 3:** MIC values for three serine protease inhibitors.

Inhibitor	Minimal Inhibitory Concentration (MIC)			
	*E. coli* 9.3 × 10^3^ cfu/mL	*S. aureus* 2.1 × 10^4^ cfu/mL	*P. aeruginosa* 1.1 × 10^4^ cfu/mL	*C. albicans* 2.1 × 10^5^ cfu/mL
**AEBSF**	2 mg/mL	2 mg/mL	0.5 mg/mL	nd
**SI**	nd	nd	nd	nd
**α_1_-AT**	nd	nd	nd	nd

nd - no biological activity was detected in the range of tested concentration.

**Table 4:** MBC/MFC values for three serine protease inhibitors.

Inhibitor	Minimal Bactericidal or Fungicidal Concentration (MBC/MFC)			
	*E. coli* 9.3 × 10^3^ cfu/mL	*S. aureus* 2.1 × 10^4^ cfu/mL	*P. aeruginosa* 1.1 × 10^4^ cfu/mL	*C. albicans* 2.1 × 10^5^ cfu/mL
**AEBSF**	4 mg/mL	3 mg/mL	3 mg/mL	nd
**SI**	nd	nd	nd	nd
**α_1_-AT**	nd	nd	nd	nd

nd - no biological activity was detected in the range of tested concentration.

**Table 5:** Summary of values for characteristic parameters describing the morphological structure of the examined biomaterials; *n* = 20.

Biomaterial	Sds	Sq	Ssk	Sku	Sdq	Sz (nm)	Ssc (1/nm)	Sdr (%)
**Uni-Graft (unmodified)**	1.545 ± 0.33 *	25.89 ± 9.96	−0.209 ± 0.73	6.3 ± 6.7	0.1659 ± 0.09	230.7 ± 104.1	0.0011 ± 0.0006	1.73 ± 1.99
**Uni-Graft + AEBSF**	1.031 ± 0.26 *	32.54 ± 11.1	0.298 ± 1.19	7.7 ± 7.7	0.1575 ± 0.16	280.0 ± 115.0	0.0009 ± 0.0005	1.38 ± 1.28
**Uni-Graft + α_1_-AT**	1.035 ± 0.35 *	34.22 ± 20.61	−0.033 ± 0.98	6.09 ± 3.96	0.1722 ± 0.09	263.6 ± 146.5	0.0010 ± 0.0005	1.76 ± 2.02

* - non-parametric Kruskal–Wallis test (*p* < 0.0001).

## Abbreviations

AEBSF: 4-(2-aminoethyl)benzenesulfonyl fluoride hydrochloride; SI: soy inhibitor; α_1_-AT: α_1_-antitrypsin; GLA: glutaraldehyde; CDI: carbodiimide; DVS: divinylsulphone

## Appendix A

**Table A1:** Characteristics of parameters describing the morphological structure of the biomaterials surface.

Parameter	Characteristics
**Sds**	Summit Density, the number of summits per unit area that make up the surface
**Sq**	Root mean square roughness
**Ssk**	Skewness of the 3D surface texture Ssk > 0—a more convex surface Ssk < 0—a more concave surface
**Sku**	Kurtosis—distribution of peaks on the surface of the material Sku = 3—normal distribution Sku > 3—inordinately high peaks Sku < 3—deep valleys
**Sdq**	General measurement of the slopes that comprise the surface
**Sz**	Characterizes the average peak to valley magnitude, which contains most of the surface heights
**Ssc**	Helps predict the degree of elastic and plastic deformation of a surface under different loading conditions
**Sdr**	Differentiates surfaces of similar amplitudes and average roughness
